# Influence of a Specific Aquatic Program on Social and Gross Motor Skills in Adolescents with Autism Spectrum Disorders: Three Case Reports

**DOI:** 10.3390/jfmk4020027

**Published:** 2019-05-24

**Authors:** Giuseppe Battaglia, Gianna Agrò, Pietro Cataldo, Antonio Palma, Marianna Alesi

**Affiliations:** 1 Department of Psychology, Educational Science and Human Movement, University of Palermo 90128 Sicily, Italy; 2 Sport and Exercise Sciences Research Unit, University of Palermo, 90144 Sicily, Italy; 3 Regional Sports School of CONI Sicilia, 90141 Sicily, Italy; 4 Department of Economics, Management and Statistics, University of Palermo, 90128 Sicily, Italy; 5 Study Center of University Sports Center of Palermo (CUS Palermo), University of Palermo, 90129 Sicily, Italy

**Keywords:** Autism Spectrum Disorders, swimming, aquatic therapy, gross motor proficiency, social skills, exercise

## Abstract

Swimming pool activities revealed to be efficacious to train psychomotor skills and increase adaptive behaviors in children with Autism Spectrum Disorders (ASD). Therefore, the purpose of this study was to investigate the efficacy of a specific multi-systemic aquatic therapy (CI-MAT) on gross motor and social skills in three adolescents with Autism Spectrum Disorders (ASD). Methods: three adolescents with ASD of which two boys (M1 with a chronological age of 10.3 years and a mental age of 4.7 years; M2 with a chronological age of 14.6 and a mental age inferior to 4 years) and one girl (chronological age of 14.0 and a mental age inferior to 4 years). The study was divided into three phases: baseline, 12-week CI-MAT program and Post-Test. Participants were administered a battery of tests incorporating anthropometric measurements, gross motor development test and a social skills questionnaire before and after a 12-week MAT-CI program. Results: Subjects improved locomotors and object control skills following the CI-MAT program in a different way. Concerning social behaviors, the higher proportion of gains was observed in the sensitivity of other’s presence and eye contact, for the contact domain, and in the comply turn for the relationship domain. Conclusions: The results of this study showed that the CI-MAT program was effective for the development of gross-motor skills and social behaviors in subjects with ASD. Moreover there is an urge to carry out a whole psychological assessment targeting both motor and adaptive development suitable to provide educational and vocational plans of exercises for people with ASD.

## 1. Introduction

Increasing research has targeted benefits of regular Physical Activity on physical and mental health in people with developmental and intellectual disabilities [1,2,3]. In this perspective, the training of motor activities to develop adaptive functioning and enhance the autonomy and the participation in social activities has been focused in individuals with Autism Spectrum Disorders (ASD) [4]. Social behaviors are very important in subjects with ASD [4]. The Interagency Autism Coordinating Committee and the National Institute of Mental Health identified the implementation of interventions to enhance social skills in subjects with ASD as a high priority. Within the typical symptomatic profile of persistent deficits in social communication and interaction across multiple contexts, children and young people with ASD showed motor impairments contributing to decrease their socialization skills [5,6]. In this population, motor diseases were rated to have a prevalence from 59–79% [7,8]. Since an early age, children with ASD showed dyspraxia, motor stereotypies, and impairment in early postural control, motor speed and coordination, balance, and fluency [9,10,11,12]. Pusponegoro et al. (2016) [13] compared gross motor proficiency endorsed by 40 children with ASD (chronological age: from 1–6 years) and 40 Typically Developing Children. They found gross motor impairments in 20% of the ASD children associated to lower socialization skills [13]. The worst performed tasks were catching a ball, riding tricycle or bicycle, and lifting and carrying. These actions became increasingly prominent with age. This revealed impaired postural control, bilateral coordination or upper-lower body coordination, and the association with lower social adaptive skills [13]. Pusponegoro and coll. [13] argued that the correlation between deficits in gross motor and socialization domains in children with ASD would be due to a failure in the activation of the mirror neurons system [13]. Mache and Todd [14] investigated the relationship between gross motor proficiency, postural stability, and restricted/repetitive behavioral patterns in a group of 11 children with ASD (5–12 years of age) compared to children without ASD. They found that the performance on gross motor tasks was influenced by the postural sway area while standing on a solid surface and children with ASD exhibited significantly greater amounts of postural sway with lower gross motor scores [14].

Given these premises, the need of early intervention to address deficits in motor skills in the ASD population is clear. However, little research has examined the physical outcomes of exercise training programs in people with ASD [3,15]. Previous study demonstrated how exercise-training programs are able to improve aerobic performance, body mass index (BMI), and muscle strength in children with moderate-to-severe forms of autism [16]. Swimming pool activities revealed to be efficacious to train psychomotor skills and increase adaptive behaviors in children with ASD [17]. Mortimer et al. (2014) in their systematic review on the beneficial effects of hydrotherapy on social and behavioral aspects in children with ASD demonstrated how structured aquatic activities, not only improve motor performances, but also provide social interaction opportunities [18]. Moreover, children with ASD enhance their self-confidence and mastery motivation when face-challenging motor tasks [17]. The positive influence of the aquatic environment can be explained as the properties of hydrostatic pressure and buoyancy that, in turn, enable improvements in sensory and social behaviors (e.g. maintaining eye contact and paying attention) and motor skills in individuals with ASD [19,20]. Despite these benefits, little studies, up to now, support these suggestions [19,20]. 

Recently, Caputo et al. [21] implemented the multi-systemic aquatic therapy (CI-MAT which corresponds to Italian Terapia Multisistemica in Acqua—TMA) aimed at decreasing social, emotional, behavioral, relational, and psychomotor deficits in people with ASD. Objectives were: to improve body posture and gestures; to cooperate during games; to recognize emotional expressions (anger, joy, shame, fear, and happiness); to decrease aggressive and self-aggressive behavior; to recognize reference people; to improve social mutuality with cooperation in social rules; to improve imitative skills; to know body scheme; to improve personal autonomy; to enhance self-esteem; to improve verbal and non-verbal communication; to decrease stereotypical behavior; and to stimulate psychomotor skills. According to the attachment theory [22], CI-MAT encourages attachment responses in the people with ASD using the primary clinging drive. During CI-MAT activities, people with ASD are induced to cling on the therapist. Recently, Caputo et al. [23] tested effectiveness of CI-MAT on behavioral, social, emotional, and swimming skills of children with ASD after a 10-month program. At post-treatment, the trained children showed significant improvements relative to controls ones on activity level, functional adaptation, emotional response, and adaptation to change [23]. Given these premises, we hypothesize that CI-MAT might also influence social and gross motor skills positively. In particular, the aim of this work was to study the effects of a CI-MAT program on locomotion (L) and object control (OC) skills and contact and interaction behaviors in three adolescents with ASD.

## 2. Materials and Methods 

### 2.1. Participants

The participants were three adolescents with ASD, of whom two were boys (M1 and M2) and one (F1) was a girl (see Table 1).

All the three subjects had been recruited through autism support groups for families and children. The inclusion criteria were: (i) to have a diagnosis of ASD which had been previously certificated by a national public health institution; (ii) to be available to attend at least 70–80% of the multi-systemic therapy in water; and (iii) to be medically able to participate in an aquatic exercise training. Subjects were excluded from the study if they had experienced orthopedic injuries or surgeries, which limited their movements, or if they did not attend at least 70–80% of CI-MAT period. Before starting the study, appropriate local ethics committee approval was obtained from the University of Palermo. Each participant’s caregiver provided signed informed consent.

All participants had been engaged in structured speech therapy and psychomotor activity since early childhood and did not attend any additional exercise training in or out of school during the experimental period. All subjects were from medium socio-economic level and lived in an urban area.

### 2.2. Procedure

The research design followed a multi-method approach for the assessment: 1. tests to measure participants’ mental age (at baseline only), anthropometric characteristics and gross motor abilities (at both baseline and post-test); 2. parental ratings of participants’ adaptive behavior (at baseline only); and 3. videos-tapes of participants’ behavior in swimming pool (at both baseline and post-test). The method of case studies was used because of its documented employ to measure the individual differences in PA interventions with individuals with ASD [24]. The study was divided into three phases: 1. baseline (bl) or Pre-Test (prt); 2. 12-week CI-MAT program; 3. Post-Test (pt). At the Pre-Test phase an assessment was carried out over six one-hour-sessions in order to measure participants’ anthropometric characteristics, gross-motor skills, mental age, adaptive and social profiles. All procedures involving human participants were in accordance with the ethical standards of the institutional and/or national research committee and with the 1964 Helsinki declaration and its later amendments or comparable ethical standards. Our methods were approved by our institution’s research council (Consiglio di Dipartimento di Scienze Psicologiche, Pedagogiche e della Formazione Prot. No. 285/2015). Written informed consent was obtained from all participants’ parents.

### 2.3. Assessment of Mental Age

The Correspondences and Functions Evaluation test (which corresponds to Italian C.F.V.—Corrispondenze e Funzioni Valutazione) [25] was administered to measure participants’ mental age. It consisted of 42 items, subdivided into five areas of logical operations: qualitative correspondences, direct quantitative correspondences, indirect quantitative correspondences, direct functions and indirect functions. For each item, evaluation was binary, with a mark of 1 being attributed to each correct item and 0 to each incorrect item. The raw data thus obtained were then transformed into a measure of mental age (range 3–14 years) on the basis of appropriate conversion tables.

### 2.4. Assessment of Adaptive Behavior 

The VABS—Vineland Adaptive Behavior Scales [26] were administered to participants’ mothers in order to measure their children adaptive development. It’s a semi-structured interview with the primary caregiver to assess adaptive behaviors necessary for everyday independent life from birth to 90 years old. It was composed of 365 items structured in four areas: Communication (receptive, expressive, and written language), Daily Living Skills (skills to take care of oneself, household, and community skills), Socialization (social relationship, emotional and behavioral regulation, and leisure activities) and Motor Skills (fine and gross motor skills). Raw scores were transformed in Age-Equivalent Scores Adjusted for Age and Level of Adaptive Functioning 

### 2.5. Assessment of Social Behaviors

An observation schedule was derived and adapted by Venuti (2001) to provide measures of participants’ interaction and contact. In detail, 8 behaviors were observed: 4 behaviors for the interaction such as joint attention, joint play, searching others’ presence, comply ones turn and 4 items for contact, such as sensitivity to other’s presence, loneliness, eye contact, observation of other’s behaviors [27].

The children were videotaped by a trained operator for 50 min during their CI-MAT session delivered by their coach in the pool and for an additional 15 min during preliminary actions in locker room.

These videos were independently rated by 2 observers (correlation among observers: more than 0.91) using the above observation schedule with scores ranging from 0 (never) to 4 (always) and measuring the rate of presence of the child’s behavior of interest. Higher scores were indicative of socially oriented behaviors except for the loneliness. 

### 2.6. Anthropometric Measurements

Anthropometric measurements were performed according to the evaluation procedures reported in several studies by Battaglia et al. [28,29] Body weight was assessed using a Seca electronic scale (maximum weight recordable 300 kg; resolution 100 g) (Seca; Hamburg, Germany) for the subjects wearing only their underwear. Height was measured by a standard stadiometer (maximum height recordable 220 cm; resolution 1 mm) with the barefoot subjects and standing upright. Body mass index (BMI) was estimated as bodyweight divided by squared height (kg/m^2^).

### 2.7. Assessment of Gross Motor Skills

The Test of Gross Motor Development (TGMD) [30] was used to measure gross motor proficiency. This is a criterion-referenced test, composed of two subtests aimed at measuring two skill sets: 7 locomotion (L) and 5 object control (OC) skills. Locomotion tasks were to run as fast as possible for 15 meters, to gallop for ten meters, to hop on one leg for five meters, to jump forward, to do a long jump, and to take little jumps forward and laterally. Object control tasks were to catch a ball with a tennis racket, to bounce off the ball, to catch a ball, to kick the ball running, and to throw a ball with the hand. Participants’ performances were videotaped with a digital video camera that allowed to analyze movement sequences separately and to assign scores. According to the handbook, participants were asked to repeat each trial three times; a score of 1 was assigned when the subject performed well twice, whilst a score of 0 was given when the subject was not able to perform the test at all. The sum of raw scores was obtained for each component and for total performance. Two locomotion and object control raw total scores (maximum total score: 48) were computed by adding the items pertaining to each scale. The Cronbach’s alpha coefficient is 0.81 and the correlation coefficient for the test-retest is 0.86.

### 2.8. Multi-Systemic Aquatic Therapy (CI-MAT) Program 

In agreement with Caputo et al. The CI-MAT protocol included three stages: Emotional Adaptation phase, Swimming Adaptation phase; Social Integration phase [23]. The CI-MAT program was performed for 12 weeks by two training sessions (45–50-min/session) per week. The “Emotional Adaptation” phase was focused on construction of the therapist-to-adolescent attachment relationship and on involvement of teenager with ASD in specific ludic water activities (dance in the water, bubbles with water and so on). Only when the adolescent performed all the ludic water activities together with the expert, the training moved on to the “Swimming Adaptation” phase. In this level the therapist coached swimming skills by adapted aquatic exercises such as: to float supine unassisted, to float prone unassisted, to glide from side to side of the pool and so on). The aim of the “Social Integration” phase was to stimulate social interaction of M1, M2 and F1 with adolescents with or without disability. In the “Emotional and Swimming Adaptation” phases, a 1:1 therapist-to-adolescent ratio was provided, instead 1:3 ratio was used during the “Social Integration” phase in order to ensure safety and optimal participation. Moreover, the validity of the therapeutic undertaking was guaranteed by the constant presence of a psychologist. 

## 3. Results

All subjects showed a Composite Scale score below the average on the Vineland Adaptive Behavior Scales (VABS) (see Table 2). 

Following the CI-MAT program subjects did not show any relevant differences in body weight, height and BMI (M1 = bl: 24.58 vs. pt: 24.75; M2 = bl: 21.41 vs. pt: 19.96; F1 = bl: 22.96 vs. pt: 20.17 kg/m^2^). As concern gross motor proficiency, subjects increased their gross motor skills from baseline to post-test in a different way (see Table 3). On a whole, M2 and F1 showed the highest improvement in their gross motor quotient (M1: bl: 6 vs. pt: 15; M2: bl: 18 vs. pt: 39; F1: bl: 14 vs. pt: 32). In detail, M1 improved almost all object control skills (items: 9–12) such as stationary bounce (9-item), catch (10-item), kick (11-item) and overhand throw (12-item) abilities; instead, as regard to locomotors ability, he only increased run (1-item) and horizontal jump skills (5-item) after the training period. M2 improved both object control (bl: 9 vs. pt: 21) and locomotors (bl: 9 vs. pt: 18) skills from baseline to post-test. F1 improved almost all locomotors skills as Run, Hop on one leg, Horizontal jump, Slip (items: 1–3, 5, 7); instead, as regard to object control ability, she only increased catch (10-item), kick (11-item) and overhand throw (12-item) skills after the training period. As shown in Table 2, the 8-item (catch a ball with a tennis racket) of object control skills revealed to be the most difficult item that did not show any significant increments in M1 and F1 after the training period. Only M2 (bl: 0 vs. pt: 3) showed relevant improvement in this skill from baseline to posttest (see Table 3).

As shown in Table 4, concerning social behaviors, the higher proportion of gains was observed in the sensitivity of other’s presence and eye contact for the contact domain and in the comply turn for the relationship domain. Furthermore, after the CI-MA program phase all subjects showed changes in their social behaviors at different levels. F1 evidenced gains in sensitivity to other’s presence (bl: 2 vs. pt: 3), eye contact (bl: 1 vs. pt: 2), joint attention (bl: 1 vs. pt: 2), joint play (bl: 2 vs. pt: 3) and comply one’s turn (bl: 1 vs. pt: 2) as well as a decrease in the frequency of loneliness behaviors (bl: 1 vs. pt: 0). M1 and M2 improved only in joint attention (bl: 1 vs. pt: 2) and comply one’s turn (bl: 1 vs. pt: 2) respectively.

## 4. Discussion

The aim of this study was to investigate the efficacy of a specific multi-systemic aquatic therapy (CI-MAT) on gross motor and social skills in three adolescents with Autism Spectrum Disorders (ASD). Results showed that the applied aquatic training program was effective to enhance object control and locomotors skills in subjects with ASD. This is coherent with previous studies that showed how exercises in water improve several aspects of gross motor proficiency, such as conditional (aerobic capacity, muscle strength, and speed) and coordination skills, in individuals with typical and atypical development [3,31]. Exercise training programs in water were found to improve aerobic capacity, physical fitness, and muscle strength in children with ASD because water gives resistance during the physical activity. Furthermore, swimming can be employed as a major component of an adapted physical activity program in ASD children. Best and Jones (1972) found that ASD children enhanced body awareness, confidence, and aquatic orientation over a 15 week swimming program [32]. 

In CI-MAT program, the water was a social activator by encouraging individuals to look for a first interaction with the CI-MAT therapist. With concern to locomotion, higher levels of improvement were found on run and hop on leg, horizontal jump, and slip. With concern to object control skills, improvements were found on almost all the tasks as catch a ball with a tennis racket, stationary bounce, catch, kick, and overhand throw. Research studies demonstrated how flexibility, balance, cardiorespiratory endurance, agility, and power increased in the child with autism after a swimming training period [15]. Yilmaz et al. (2004) [15] showed that a swimming exercise training was effective to develop physical fitness and aquatic orientation in children with ASD. Object control findings could be due to the relationship among perceptual ability, visual-motor integration and motor proficiency. Water exercise could be suitable to stimulate this relationship. To perform complex motor patterns, such as object control ones, a subject is asked to process afferent information in a rapid and efficient way [33,34]. Moreover, training sessions stimulated the participation to CI-MAT as almost all subjects attended at least 70–80% of the CI-MAT program. M2, instead, attended almost all the training period and after the experimental period was involved in CI-MAT sessions/week with other five persons with a therapist-subject ratio of 1:6. This subject increased in a significant way his locomotors (baseline score: 9 vs. posttest score: 21; Δ = 133.33%) and object control (baseline: 9 vs. posttest: 18; Δ = 100%) skills after the CI-MAT program period. Moreover, M2 ranked second in a swimming race on the 25-meter freestyle of the Federazione Italiana Sport Disabilità Intellettiva Relazionale (Fisdir) with a time of 36 min and 6 s. As concern social behaviors, almost all subjects seemed to have enhanced their skills from baseline to post-test. Specifically, F1 improved social skills more than others and showed gains both in contact and interaction. This result needs to be related to her mental age because F1 showed the highest mental age (6.9 year). M1 and M2 revealed to gain less social improvements from baseline to post-test by increasing only joint attention and comply one’s turn, respectively. Even though they had different chronological age, they had similar mental age (4.3 and 4.7 year). Considering all the results together concerning motor and social skills, F1 again improved more than others. Her performance on locomotors skill tasks significantly increased by 133% as well as on object control tasks improved by 120%. This result is consistent with the hypothesis that the efficacy of motor programs would be influenced by the level of mental age. Individuals with higher mental age would improve more than those with a severe level of ID after specific motor trainings [35]. In our study, we observed gender bias. These has been repeatedly observed in neuropsychiatric disorders. In particular, several studies in schools have shown a 30–50% excess of males over females in people with intellectual disabilities. In autism spectrum disorder (ASD), the male:female ratio is 4:1. It drops to 2:1 for individuals with moderate to severe intellectual disabilities, and increases to 7:1 for high-functioning autism [36].

The socialization was a crucial element of CI-MAT program in children with ASD. In particular, a special interaction between each participant and the trainer was recorded on the eye contact, waiting times, cooperation during games, and paying attention. For this the CI-MAT program was performed in a public swimming pool to promote the socializing of participants with their peers. A public structured environment, revealed to be a suitable therapeutic setting during the experimental period.

## 5. Implications

On the whole, findings contribute additional evidence that suggests the effectiveness of beneficial effects of aquatic activities on motor and social skills supporting the hypothesis that motor and intellectual domains are highly interrelated in individuals with atypical development. As a consequence, there is an urge to plan whole psychological assessment targeting both motor and adaptive development suitable to direct the implementation of educational and vocational plans of exercises for people with ASD. However, shortcomings of the current research limit findings generalizability: firstly, we do not have a control group with typical development and we did not control other physical or mental activities. Secondly, it would be interesting to carry out a follow up over 3 months to assess the maintenance of obtained improvements. New research is needed to investigate the effects of the water-training program with more large samples.

## Figures and Tables

**Table 1:** Characteristics of the subjects.

	M1	M2	F1
**Chronology Age** (CA)	11 y	14.8 y	15.11 y
**Mental Age** (MA)	4.3 y	4.7 y	6.9 y
**Height** (h)	1.39 m	1.66 m	1.57 m
**Weight** (W)	47.5 kg	59.0 kg	56.6 kg
**Body mass index** (BMI)	24.58	21.41	22.96

**Table 2:** Participants’ adaptive development.

			M1	M2	F1
**VABS**	**Composite Scale index**	CS	1.7 y	2.4 y	2.4 y
	**Sub-test VABS**	C	2.10 y	3.7 y	2.10 y
		DS	2.10 y	2.8 y	2.10 y
		S	2.6 y	2.0 y	2.6 y
		MS	<1.6 y	<1.6 y	<1.6 y

Legend: C = Communication; DS = Daily Skills; S = Socialization; MS = Motor Skills.

**Table 3:** Evaluation of locomotors and object control skills after the multi-systemic therapy program in water.

	Item	Times	Subject		
			M1	M2	F1
**Locomotors Skills**	**1**	*Baseline*	1	4	2
		*Posttest*	3	4	4
	**2**	*Baseline*	0	0	1
		*Posttest*	0	2	4
	**3**	*Baseline*	0	0	0
		*Posttest*	0	4	4
	**4**	*Baseline*	0	0	0
		*Posttest*	0	1	0
	**5**	*Baseline*	1	2	2
		*Posttest*	2	4	4
	**6**	*Baseline*	0	0	1
		*Posttest*	0	2	1
	**7**	*Baseline*	0	3	3
		*Posttest*	0	4	4
	***Score***	*Baseline*	2	9	9
		*Posttest*	5	21	21
**Object Control Skills**	**8**	*Baseline*	0	0	0
		*Posttest*	0	3	0
	**9**	*Baseline*	0	1	2
		*Posttest*	2	3	2
	**10**	*Baseline*	2	4	2
		*Posttest*	4	4	4
	**11**	*Baseline*	1	2	0
		*Posttest*	2	4	1
	**12**	*Baseline*	1	2	1
		*Posttest*	2	4	4
	***Score***	*Baseline*	6	18	14
		*Posttest*	15	39	32

Legend: 1. Run; 2. Gallop; 3. Hop on one leg; 4. Leap; 5. Horizontal jump; 6. Skip; 7. Slip; 8. Catch a ball with a tennis racket; 9. Stationary bounce; 10. Catch; 11. Kick; 12. Overhand Throw.

**Table 4:** Frequencies of social behaviors targeting contact and interaction before and after the aquatic adapted physical activity program.

	Item	Times	Subject		
			M1	M2	F1
**Contact**	**1**	*Baseline*	2	2	2
		*Posttest*	1	2	3
	2 *	*Baseline*	1	0	1
		*Posttest*	0	0	0
	**3**	*Baseline*	1	1	1
		*Posttest*	1	1	2
	**4**	*Baseline*	2	1	1
		*Posttest*	1	1	1
**Interaction**	**5**	*Baseline*	1	2	1
		*Posttest*	2	2	2
	**6**	*Baseline*	2	2	3
		*Posttest*	2	3	2
	**7**	*Baseline*	0	0	1
		*Posttest*	0	0	1
	**8**	*Baseline*	1	1	1
		*Posttest*	1	2	2

Legend: 1. Sensitivity to other’s presence; 2. Loneliness; 3. Eye contact; 4. Observation of other’s behaviors; 5. Joint attention; 6. Joint play; 7. Searching others’ presence; 8. Comply ones turn. Note: 0: never; 1: rarely; 2: sometimes; 3: often; 4: always. * Only for 2. Loneliness 0: always; 1: often; 2: sometimes; 3: rarely; 4: never.
